# Revisiting Technology’s Impact: A Pre-and During-COVID Study on Mental Health in Older Adults

**DOI:** 10.1093/geroni/igaf122.2713

**Published:** 2025-12-31

**Authors:** Geunhye Park, Jeonghun Kim

**Affiliations:** SUNY Cortland, Cortland, New York, United States; SUNY Cortland, Cortland, New York, United States

## Abstract

The COVID-19 pandemic has exacerbated mental health challenges among older adults, yet existing research remains methodologically limited, often relying on cross-sectional designs that fail to capture temporal changes. This study aims to fill this gap by examining individual-level mental health trajectories before and during the pandemic, contributing to deeper understanding of the long-term psychological effects of crisis events on older populations. Using a longitudinal fixed-effects probit model, we analyze data from the National Health and Aging Trends Study (NHATS) Round 9 (pre-pandemic) and Round 10 (during the pandemic), allowing us to control for unobserved heterogeneity and incorporate lagged variables that account for delayed mental health effects. The sample includes 3,257 respondents, predominantly female (58%), White American (76%), and aged 75-79 (29%). Findings reveal that increased technology use-particularly virtual communication, telehealth, and online social engagement-was associated with greater mental health deterioration among older adults. However, these effects were contingent on pre-existing mental health conditions, as those with a history of depression or anxiety experienced negative impacts. Additionally, income level emerged as a stronger predictor of mental health outcomes than technology use, particularly among individuals with severe mental health conditions, highlighting economic security as a critical determinant of resilience. These findings contribute to theoretical discussions on crisis-induced vulnerability and have significant policy and clinical implications. Policymakers must strengthen income support programs, social safety nets, and digital inclusion efforts, while clinicians should recognize that technology-driven interventions must be accompanied by financial and mental health support to effectively address disparities among older adults.

